# Variation in Adult Outpatient Opioid Prescription Dispensing by Age and Sex — United States, 2008–2018

**DOI:** 10.15585/mmwr.mm6911a5

**Published:** 2020-03-20

**Authors:** Lyna Z. Schieber, Gery P. Guy, Puja Seth, Jan L. Losby

**Affiliations:** 1 Division of Unintentional Injury Prevention, National Center for Injury Prevention and Control, CDC.

In 2017, prescription opioids were involved in 36% of opioid-involved overdose deaths in the United States (*1*). Prescription opioids can be obtained by prescription or through diversion (the channeling of regulated drugs from legal to illegal sources) (*2*). Among new heroin users, 66%–83% reported that their opioid use began with the misuse of a prescription opioid (*3*). “Misuse” is generally defined as drugs taken for a purpose other than that directed by the prescribing physician, in greater amounts, more often, or for a longer duration than prescribed (*2*). Exposure to prescription opioids can be lessened by ensuring recommended prescribing, thereby potentially reducing the risk for misuse, opioid use disorder, and overdose (*4*). Sex and age groups with high exposure to prescription opioids are not well defined. Using a retail pharmaceutical database from IQVIA,* nationwide trends in opioid prescription fill rates for adult outpatients by age and sex were examined during 2008–2018. Opioid prescription fill rates were disproportionately higher among men and women aged ≥65 years and women of all ages. For reasons not well understood, these disparities persisted over 11 years even as the opioid fill rate declined for each age group and sex. Interventions to improve prescribing practices by following evidence-based guidelines that include weighing the benefits and risks for using prescription opioids for each patient and adopting a multimodal approach to pain management could improve patient safety while ameliorating pain. These efforts might need to consider the unique needs of women and older adults, who have the highest opioid prescription fill rates.

The IQVIA administrative database Total Patient Tracker was used to identify patients aged ≥20 years who had at least one opioid prescription filled in a given year during January 1, 2008–December 31, 2018. A second IQVIA database (SMART—Patient Insights) was used to determine the total number of opioid prescriptions filled each year. These databases recorded information from approximately 50,400 retail pharmacies, representing 92% of all U.S. retail prescriptions. Data were weighted to provide nationwide estimates. Prescriptions written by veterinarians or oncologists were excluded to avoid including prescriptions for animals or for human patients undergoing active cancer treatment, as were records for which age or sex was unknown (approximately 2.0% each). Data were not available from mail order prescription services, or from prescriptions provided directly by prescribers or at methadone maintenance treatment clinics. Cough or cold formulations containing opioids and buprenorphine products commonly used to treat opioid use disorder were also excluded. Because only existing, deidentified data were used, CDC determined the study to be exempt from human subject regulations and institutional review board review.

To compute the age-standardized annual percentage of the U.S. adult population aged ≥20 years with a filled opioid prescription, the number of all unique persons who had an opioid prescription filled in a given year was divided by the estimated U.S. census population during that year for each respective age group. Pearson’s chi-squared test of categorical data was used to test for differences in annual percentage distributions among age groups and sex using SAS (version 9.4; SAS Institute). Temporal trends during 2008–2018 were assessed by fitting log-linear regression models and comparing trends among groups by pairwise comparison parallel or coincidence testing using Joinpoint regression software (version 4.5.0.1; National Cancer Institute). All hypothesis testing was two-tailed, using p<0.05 to indicate statistical significance.

In 2018, an opioid prescription was filled by 19.2% of the adult U.S. population, with an average of 3.6 prescriptions per patient (Table). Among adults aged ≥65 years, 25.0% had at least one opioid prescription filled in 2018, including 23.5% of men and 26.1% of women. Compared with patients aged 20–24 years, those aged ≥65 years were approximately 2.6 times as likely to have had an opioid prescription filled in 2018 (25.0% versus 11.2%; odds ratio [OR] = 2.64; 95% confidence interval [CI] = 2.63–2.65; p<0.001).

**TABLE:** Trends in the annual percentage*of adults aged ≥20 years who had an opioid prescription filled, by age group and sex — United States, 2008–2018

Sex/Age group (yrs)	Patients with at least one opioid prescription filled						% Change from 2008 to 2018	AAPC (95% CI) from 2008 to 2018**^§^**
	2008			2018				
	No. (%)*	OR (95% CI)	Opioid prescription per patient^†^	No. (%)*	OR (95% CI)	Opioid prescription per patient^†^		
**Men and women**								
**Total^¶^**	**60,954,146 (27.8)**	**N/A**	**3.6**	**47,504,970 (19.2)**	**N/A**	**3.6**	**−31**	**−3.5 (−4.9 to −2.1)**
20–24	4,755,234 (22.5)	Referent	2.0	2,468,395 (11.2)	Referent	1.7	**−**50	**−**6.7 (**−**7.5 to **−**5.9)
25–34	11,000,783 (27.4)	1.30 (1.29 to 1.31)**	2.7	6,786,718 (14.8)	1.37 (1.36 to 1.38)**	2.5	**−**46	**−**5.9 (**−**7.3 to **−**4.5)
35–44	11,466,903 (27.2)	1.29 (1.28 to 1.30)**	3.4	7,417,100 (17.9)	1.73 (1.72 to 1.74)**	3.4	**−**34	**−**3.9 (**−**5.3 to **−**2.6)
45–54	12,989,778 (29.2)	1.42 (1.41 to 1.43)**	4.2	8,547,366 (20.4)	2.04 (2.03 to 2.05)**	4.0	**−**30	**−**3.3 (**−**4.2 to **−**2.4)
55–64	9,843,599 (28.8)	1.39 (1.38 to 1.40)**	4.1	10,184,432 (23.9)	2.49 (2.48 to 2.50)**	4.5	**−**17	**−**1.7 (**−**2.3 to **−**1.0)
≥65	11,463,550 (29.6)	1.45 (1.44 to 1.46)**	3.8	13,177,942 (25.0)	2.64 (2.63 to 2.65)**	3.8	**−**16	**−**1.7 (**−**2.3 to **−**1.0)
**Men**								
**Total^¶^**	**25,415,537 (23.8)**	**Referent**	**3.4**	**19,819,894 (16.5)**	**Referent**	**3.7**	**−31**	**−3.5 (−6.6 to −0.4)**
20–24	1,828,929 (16.9)	Referent	1.9	925,544 (8.2)	Referent	1.7	**−**51	**−**6.5 (**−**8.0 to **−**5.0)
25–34	4,341,681 (21.5)	Referent	2.6	2,491,609 (10.6)	Referent	2.9	**−**51	**−**6.7 (**−**8.6 to **−**4.7)
35–44	4,884,731 (23.3)	Referent	3.3	3,010,659 (14.5)	Referent	3.5	**−**38	**−**4.4 (**−**6.2 to **−**2.7)
45–54	5,749,176 (26.3)	Referent	4.1	3,677,678 (17.8)	Referent	4.0	**−**32	**−**3.6 (**−**4.7 to **−**2.6)
55–64	4,376,831 (26.6)	Referent	4.0	4,612,416 (22.4)	Referent	4.5	**−**16	**−**1.5 (**−**2.2 to **−**0.8)
≥65	4,434,694 (26.7)	Referent	3.4	5,531,474 (23.5)	Referent	3.6	**−**12	**−**1.2 (**−**2.3 to **−**0.0)
**Women**								
**Total^¶^**	**35,538,609 (31.5)**	**1.45 (1.44 to 1.46)^††^**	**3.7**	**27,685,077 (21.9)**	**1.45 (1.44 to 1.46)^††^**	**3.6**	**−30**	**−3.2 (−4.5 to −2.0)**
20–24	2,926,305 (28.3)	1.95 (1.94 to 1.96)^††^	2.0	1,542,851 (14.3)	1.88 (1.87 to 1.89)^††^	1.6	**−**49	**−**7.0 (**−**7.5 to **−**6.4)
25–34	6,659,102 (33.0)	1.79 (1.78 to 1.80)^††^	2.7	4,295,109 (19.0)	1.97 (1.96 to 1.98)^††^	2.4	**−**42	**−**5.3 (**−**6.6 to **−**4.0)
35–44	6,582,172 (31.1)	1.49 (1.48 to 1.50)^††^	3.5	4,406,440 (21.3)	1.58 (1.57 to 1.59)^††^	3.2	**−**32	**−**3.6 (**−**4.8 to **−**2.4)
45–54	7,240,602 (32.0)	1.32 (1.31 to 1.33)^††^	4.3	4,869,688 (23.0)	1.38 (1.37 to 1.39)^††^	4.0	**−**28	**−**3.0 (**−**3.8 to **−**2.3)
55–64	5,466,768 (30.9)	1.24 (1.23 to 1.25)^††^	4.2	5,572,016 (25.3)	1.17 (1.16 to 1.18)^††^	4.5	**−**18	**−**1.8 (**−**2.4 to **−**1.2)
≥65	7,028,856 (31.7)	1.27 (1.26 to 1.28)^††^	4.1	7,646,467 (26.1)	1.23 (1.22 to 1.24)^††^	4.0	**−**18	**−**1.8 (**−**3.2 to **−**0.5)

**Abbreviations:** AAPC = average annual percentage change; CI = confidence interval; N/A = not applicable; OR = odds ratio.

* Percentages are age-adjusted to the 2000 U.S. census population.

^†^ Calculated by totaling the number of opioid prescriptions and dividing by the total number of patients who received at least one opioid prescription in a study year.

^§^ Indicates that AAPC was significantly different from zero at the alpha = 0.05 level.

^¶^ The numbers by age groups do not sum to the total number of all adults aged ≥20 years in each study year because the total number was calculated for patients aged ≥20 years from Total Patient Tracker to avoid potential double-counting of persons who progress in age; these patient numbers are weighted estimates.

** Indicates Pearson’s chi-squared test was significant (p<0.001) compared with those aged 20–24 years who had an opioid prescription filled or not.

^††^ Indicates Pearson’s chi-squared test was significant (p<0.001) compared with their male counterpart in the same age group who had an opioid prescription filled or not.

From 2008 to 2018, the percentage of adults who had an opioid prescription filled declined 31% overall, from 27.8% to 19.2%, an average of 3.5% per year (95% CI = −4.9% to −2.1%; p<0.001). This decline was significant for each age group and sex (Figure 1) (Table). The magnitude of decline varied fourfold by age group, ranging from 1.7% each year among patients aged 55–64 and ≥65 years (95% CI = −2.3% to −1.0%; p<0.001) to 6.7% among patients aged 20–24 years (95% CI = −7.5% to −5.9%; p<0.001) (Table).

**FIGURE 1:**
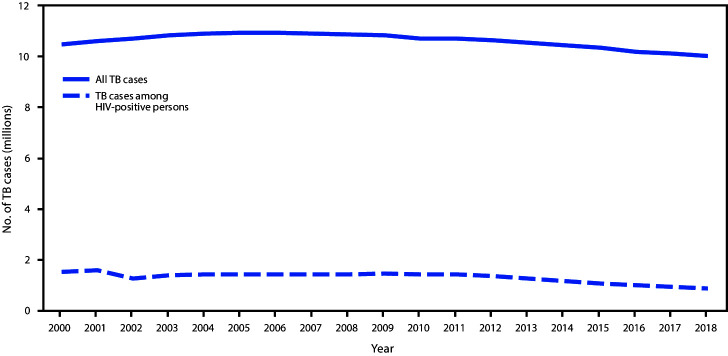
Comparison of trends in the annual percentage of adults aged ≥20 years who had an opioid prescription filled, by age group and sex — United States, 2008–2018 * Indicates that average annual percentage change during 2008–2018 was significantly different from zero at the alpha = 0.05 level by using Joinpoint regression analysis. ^†^ Indicates that two trends in terms of average annual percentage change compared between men and women of the same age group were parallel and identical, using parallelism or coincidence test that examines whether two regression mean functions (slope of the change in trend) are similar or identical in direction at p<0.05.

For each age group, a statistically higher percentage of women than men filled at least one opioid prescription over the 11-year study period (Figure 2). In 2018, women had approximately 1.5 times the odds of filling an opioid prescription overall than did men (21.9% versus 16.5%; OR = 1.45; 95% CI = 1.44–1.46; p<0.001) (Table). Within each age group, the odds among women were significantly higher than were those among men. This difference was largest among persons aged 25–34 years, among whom women had nearly twice the odds of filling an opioid prescription than did men (19.0% versus 10.6%; OR = 1.97; 95% CI = 1.96–1.98; p<0.001).

**FIGURE 2:**
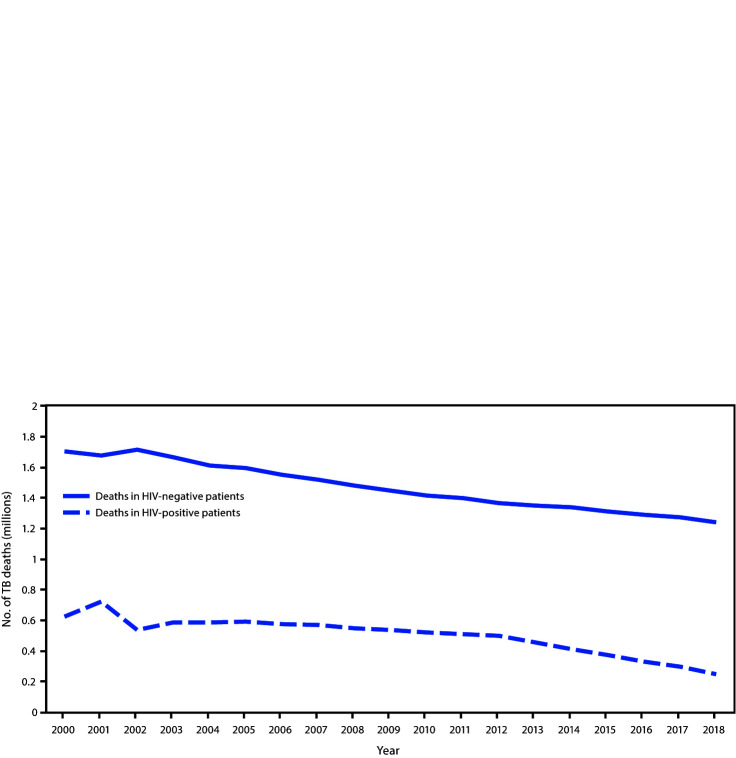
Trends in odds of women having an opioid prescription filled compared with men, by age group among adults aged ≥20 years — United States, 2008–2018 * Indicates Pearson’s chi-squared test was significant (p<0.001) for differences in annual percentage distributions among each age group and sex each year during 2008–2018.

## Discussion

The annual percentage of U.S. adults who had an opioid prescription filled decreased by 31% during 2008–2018. This decline might be attributed to implementation of several opioid prescribing guidelines, enhanced prescription drug monitoring programs, and other quality improvement initiatives (*5*). Percentages of persons with at least one opioid prescription filled were the highest among adults aged ≥65 years. These persons might have higher frequency, longer duration, or greater intensity of chronic pain, which might contribute to higher prescription fill rates (*6*). Some researchers have described a stable trend from 2007 to 2016 among commercially insured and Medicare Advantage beneficiaries in opioid prescription fill rates (*7*), whereas the findings in this study indicated a decline. Although the reasons for this discrepancy are not clear, the patient population of the current study is different from that of the study of Medicare Advantage beneficiaries and includes all classes of payers.

Higher opioid prescription fill rates among older adults is particularly worrisome because they are more likely to have an adverse event, even death, from taking an opioid medication (*8*). Older adults might also be less aware of the number of doses taken, have problems with balance or gait, experience a drug interaction with another medication used to treat a chronic condition, or have reduced opioid excretion resulting from age-related changes in liver and renal function (*8*). The percentage decline of opioid prescriptions filled by patients aged ≥65 years was the smallest of any age group, only 16% over 11 years.

Compared with men, women in all age groups had higher odds of having an opioid prescription filled. This might be partly explained by physical differences in how women process pain (*9*), higher likelihood of having a diagnosis of a mental health disorder, greater use of health care, or higher prevalence of certain chronic health conditions for which opioids are commonly prescribed (e.g., arthritis and fibromyalgia) compared with that of men (*10*). In addition, younger women might receive opioids during their childbearing years for painful reproductive disorders (e.g., dysmenorrhea or endometriosis) (*10*). However, the extent to which these conditions are driving these differences is unknown.

The findings in this report are subject to at least five limitations. First, only those prescriptions filled by retail pharmacies were considered; data were not available from other sources. Second, analyzing dosage, duration, or type of formulation was beyond the scope of this study. Third, information was not available on prescriptions that were written but not filled, whether any or all of the prescription was taken by the patient, and whether the prescription was new versus a refill. Fourth, this report did not assess drug diversion, which could result in prescription opioids being obtained through illicit sources (*2*). Finally, the efficacy of the prescription relative to the medical condition and severity could not be determined.

Those age groups among both sexes with the highest prescription fill rates warrant special attention to understand whether and how prescribing might be reduced. Optimal prescribing for these groups might differ from that of other groups because best practices for treating pain vary by medical condition and pharmacokinetics, and the prevalence of medical conditions varies by age group and sex (*4*). Additional research could help better identify patient needs and effective multimodal approaches to pain management, particularly among women and persons aged ≥65 years, the groups with higher opioid prescription fill rates. This in turn could help to establish the extent to which the observed differences in fill rates are relevant and lead to optimal prescribing for all subpopulations.

**Summary**

**What is already know about this topic?**

One third of U.S. opioid overdose deaths in 2017 involved prescription opioids despite reductions in opioid dispensing since 2012. Sex and age groups with high exposure to prescription opioids are not well defined.

**What is added by this report?**

One in five adults had an opioid prescription filled in 2018, with higher fill rates among women than men across age groups. Although fill rates declined in each age group among both sexes during 2008–2018 (31% overall), disparities persisted. Rates among adults aged ≥65 years were highest and declined least.

**What are the implications for public health practice?**

Efforts to improve opioid prescribing need to consider the unique needs of women and older adults while using multimodal approaches to pain management.

## Footnotes

*
https://www.iqvia.com/locations/united-states/solutions/commercial-operations/essential-information/prescription-information .
